# Association between platelet-to-albumin ratio and total spine bone mineral density in older adults with thoracolumbar fragility fractures: a retrospective cross-sectional study

**DOI:** 10.3389/fendo.2026.1717807

**Published:** 2026-02-11

**Authors:** Xinhuan He, Weicong Yin, Dixi Huang, Chuqun Chen, Weiqiang Lai, Siyuan Hu, Xu Li, Kaiqin Gong, Kunrui He, Weile Liu, Shoubin Huang, Shaowei Zheng, Jianping Zheng, Chunhan Sun, Guowei Zeng

**Affiliations:** Department of the Orthopaedic, Huizhou First Hospital, Guangdong Medical University, Huizhou, Guangdong, China

**Keywords:** albumin, bone mineral density, osteoporosis, platelets, platelet-to-albumin ratio, thoracolumbar fragility fractures

## Abstract

**Background:**

Global aging elevates the incidence of thoracolumbar fragility fractures (TLFFs), which are linked to osteoporosis. Platelets (PLT) and albumin influence bone health; the platelet-to-albumin ratio (PAR) is applied in multi-disease research, but it has not yet been applied to patients with TLFF. This study thus explores PAR’s association with bone mineral density (BMD).

**Methods:**

This retrospective cross-sectional analysis based on a cohort of TLFF patients included 703 elderly TLFF patients from Huizhou First Hospital (2020–2025). BMD was measured, PAR calculated, and grouped; covariates were collected. Subsequently, characterization of the study population, univariate linear regression analyses, and subgroup as well as interaction analyses were carried out.

**Results:**

High-PAR groups had higher PLT, lower albumin, and lower BMD. PAR showed a significant negative association with total spine BMD (TS-BMD) even after adjustment; the initial association of neutrophill-to-albumin ratio (NPAR) became non-significant post-adjustment.

**Conclusion:**

In elderly TLFF patients, PAR is negatively associated with TS-BMD. As an easily measurable index, PAR may act as a clinical tool.

## 1 Introduction

Societal progression has fueled a global demographic shift toward aging populations, concurrently driving a steady increase in the incidence of thoracolumbar fragility fractures (TLFFs)—a condition tightly linked to osteoporosis and a growing concern in orthopedic medicine (1, 2). As a devastating sequela of osteoporosis, TLFFs are distinguished not only by their high prevalence but also by the heavy burdens of mortality and long-term disability they impose (3). Together, these characteristics pose an urgent challenge to global public health systems and day-to-day clinical orthopedic practice (4).

For patients with TLFFs unable to tolerate conservative management, prolonged bed rest is a primary concern. This practice is inherently associated with heightened risks of complications such as venous thromboembolism, accelerated bone loss, and sarcopenia—all of which further impair patient prognosis. In such cases, minimally invasive vertebral augmentation techniques, specifically percutaneous vertebroplasty (PVP) (5) and percutaneous kyphoplasty (PKP) (6), have emerged as well-validated therapeutic modalities in contemporary clinical orthopedics (7). The primary objectives of these interventions are to achieve rapid pain relief and facilitate early ambulation; these two pivotal endpoints directly mitigate the risk of secondary complications and improve overall patient outcomes, spanning functional recovery to long-term survival (8). Importantly, the severity of underlying osteoporosis strongly correlates with fracture risk, including both index TLFFs and recurrent fractures (9). Developing multi-dimensional, targeted strategies to ameliorate osteoporosis in patients with a history of prior fractures—particularly those who have undergone surgical intervention—remains a critical unresolved challenge in orthopedic practice (10).

Marrow-resident megakaryocytes play a pivotal role in skeletal homeostasis by regulating both bone formation and bone resorption (11, 12). Prior clinical investigations have established a correlation between serum albumin concentration and the severity of osteoporosis. For instance, Zhao et al. identified low albumin levels as an independent risk factor for osteoporosis. Notably, albumin status also warrants consideration when managing glycemic control in patients with T2DM (13). Complementarily, Yoshio Nagayama et al. demonstrated that reduced serum albumin concentrations are significantly and independently linked to a higher prevalence of osteoporosis (14). As the most abundant protein in the circulatory system, albumin (ALB) serves a range of physiological functions that include sustaining colloid osmotic pressure, facilitating molecular transport, and conferring anti-inflammatory, antioxidant, and endothelial protective effects. Importantly, hypoalbuminemia is closely linked to a spectrum of metabolic disturbances, such as malignant tumors, nephrotic syndrome, and inflammatory-malnutrition syndrome (15).

The PAR—a composite index integrating platelet counts and albumin levels—has been extensively applied in clinical research across multiple disease domains, including cardiovascular disorders, endocrine conditions, pulmonary diseases, kidney disease, and malignancies (16–20). To the present day, no studies have documented an association between the PAR and osteoporosis severity in patients with thoracolumbar fractures. Currently, BMD is most commonly used in clinical practice to evaluate the severity of osteoporosis.

Accordingly, this retrospective cross-sectional study collected clinical data from elderly patients with thoracolumbar fractures admitted between 2020 and 2025, aiming to explore the potential relationship between PAR and BMD.

## 2 Materials and methods

### 2.1 Study population

**Figure 1:**
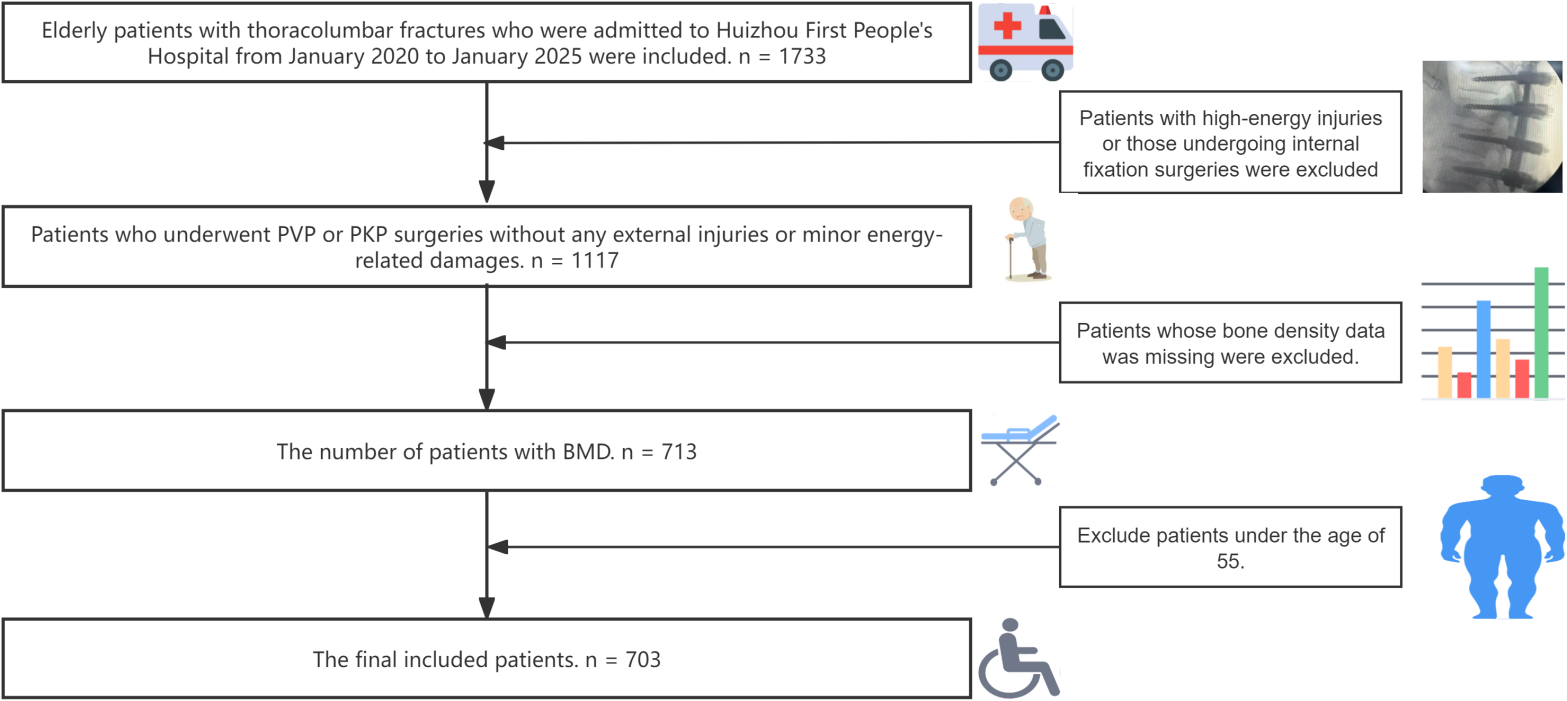
Inclusion and exclusion flowchart.

We conducted a retrospective cross-sectional analysis analysis of patients who received treatment for thoracolumbar fractures at Huizhou First Hospital from 2020 to 2025. We enrolled eligible patients based on the following inclusion criteria (1): Patients with a clear diagnosis of thoracolumbar fragility fractures who have received PVP or PKP treatment (2). Elderly patients over 55 years old. The following patients need to be excluded: (1)Those who have not undergone bone density testing or have lost spinal bone mineral density data (2). Patients with thoracolumbar burst fractures or high-energy injury fractures or those undergoing internal fixation surgeries. As this study was based on real-world clinical research, no screening for previous underlying diseases was conducted, which can make the outcome closer to the real world. Ultimately, a sum of 703 patients was incorporated into our research. The detailed process can be found in **Figure 1**.

This study was approved by the Ethics Committee of the Huizhou First Hospital and conducted in accordance with the Declaration of Helsinki (KYLL-2025-146-01). Given its retrospective design, the Committee granted a waiver of written informed consent. As this was a retrospective cross-sectional analysis, the Ethics Committee approved the application for a waiver of signed informed consent. Moreover, the study’s findings will be shared with participants and applied in clinical trials to monitor any potential clinical advantages. The interventions used in this study are examinations that are already accessible to patients during their hospital stays, and they do not entail any extra costs for the patients. To protect patient privacy, this study anonymized the identifiable information of the enrolled patients.

### 2.2 Covariates

Considering that additional factors may influence osteoporosis indices, covariates were included in the analyses. These covariates were selected based on prior literature. The variables incorporated in the present study covered three core categories: demographic characteristics, clinical history, and laboratory parameters. Demographic characteristics included gender, age, height, weight, body mass index (BMI), and blood type; clinical history encompassed trauma history, known osteoporosis status, and the presence of comorbidities such as hypertension, diabetes mellitus, pulmonary disease, renal disease, and a history of tumors; laboratory parameters further included hematological indices and biochemical indices—among which hematological indices comprised erythrocyte sedimentation rate (ESR), C-reactive protein (CRP), hemoglobin concentration, red blood cell count(RBC), white blood cell count(WBC), neutrophil count(NC), lymphocyte count(LYM), monocyte count(MC), and platelet count(PLT), while biochemical indices involved fasting blood glucose, serum electrolytes (sodium, potassium, chloride, calcium), renal function markers (urea, creatinine, uric acid), lipid profiles (total cholesterol, triglycerides), serum proteins (albumin, globulin, albumin/globulin ratio), and liver function markers (total bilirubin, direct bilirubin), and BMD. Detailed information regarding all the aforementioned variables is provided in **Table 1**. Given the presence of missing values in certain datasets of this study, we employed IBM SPSS Statistics software to perform missing value imputation. The supplementary data can be seen in **Table 2**. The formulae used were: PAR=PLT/albumin, NPAR=NC × PLT/albumin.

**Table 1:** Baseline characteristics of elderly patients with thoracolumbar fractures were included in the final analysis.

Characteristics			Mean or proportion	
	N			p
Age[year], mean	703		75.94 ± 8.71	**0.006**
Gender, n(%)	703	Male	115(16.36%)	**<0.001**
		Female	588(83.64%)	
Height[cm], mean	445		155.14 ± 8.03	**<0.001**
Weight[kg], mean	445		53.84 ± 10.41	**<0.001**
BMI[kg/m^2^], mean	445		22.3 ± 3.68	**<0.001**
Drug allergy, n (%)		Yes	686 (97.58%)	0.252
		No	17 (2.42%)	
Traumatism, n (%)	706	Yes	293(41.68%)	**<0.001**
		No	410(58.32%)	
Osteoporosis, n (%)	703	Yes	527(74.96%)	**0.001**
		No	176(25.04%)	
Hypertension, n (%)	703	Yes	274(38.98%)	**0.001**
		No	429(61.02%)	
Diabetes, n (%)	703	YES	83 (11.81%)	**<0.001**
		No	620 (88.19%)	
Pulmonary disease, n (%)	703	Yes	55 (7.82%)	0.256
		No	648 (92.18%)	
Kidney disease, n (%)	703	Yes	10 (1.42%)	**0.002**
		No	693 (98.56%)	
Tumor history, n (%)	703	Yes	31 (4.41%)	**0.004**
		No	672 (95.59%)	
Erythrocyte sedimentation rate(ESR), mean	393		39 ± 25.02	0.677
C-reactive protein(CRP), mean	257		18.43 ± 26.34	0.133
Hemoglobin(HGB)[g/dL], mean	693		120.66 ± 17.9	**<0.001**
Red blood cell(RBC)[×10^9^/L], mean	693		4.24 ± 0.67	0.433
White blood cell(WBC)[×10^12^/L], mean	693		7.35 ± 2.67	**0.015**
Neutrophil cell(NC)[×10^9^/L], mean	693		5.08 ± 2.48	**0.027**
Lymphocyte cell(LC)[×10^9^/L], mean	693		1.48 ± 0.61	0.579
Monocyte cell(MC)[×10^9^/L], mean	693		11.04 ± 25.84	**0.029**
Platelet(PLT)[×10^9^/L], mean	693		240.65 ± 77.89	**<0.001**
Fasting blood-glucose(FBG)[mm/h], mean	693		6.2 ± 2.55	**0.001**
Natrium(Na^2+^)[mmol/L], mean	692		140.98 ± 2.8	0.213
Kalium(K^+^)[mmol/L], mean	692		3.89 ± 0.43	0.391
Chloridion(Cl^-^)[mmol/L], mean	692		104.94 ± 3.2	0.794
Calcium(Ca2+)[mmol/L], mean	692		2.25 ± 0.14	0.066
Urea[mmol/L], mean	693		6.74 ± 3.36	**0.018**
Serum creatinine[umol/L], mean	693		76.33 ± 75.58	**0.001**
Uric acid(Ua)[μmol/L], mean	492		334.41 ± 107.85	**<0.001**
Total cholesterol(TC)[mmol.L], mean	458		4.71 ± 1.1	0.136
Triglyceride(TG)[mmol.L], mean	458		1.37 ± 0.69	**0.034**
Albumin(ALB)[g/L], mean	690		37.56 ± 4.64	0.317
Globulin(GLB)[g/L], mean	690		32.94 ± 5.89	0.256
Albumin/Globulin(AG), mean	690		1.18 ± 0.26	0.124
Total bilirubin(TBil)[μmol/L], mean	633		12.9 ± 8.59	0.148
Direct bilirubin(DBil)[μmol/L], mean	588		4.77 ± 3.14	0.162
L2-BMD, mean	703		-3.71 ± 1.16	**<0.001**
L3-BMD, mean	703		-3.81 ± 1.49	**<0.001**
L4-BMD, mean	703		-3.51 ± 1.29	**<0.001**
TS-BMD, mean	703		-3.68 ± 1.24	**<0.001**
Platelet-to-Albumin Ratio(PAR, × 10^9^/g), mean	689		6.51 ± 2.37	**0.006**
Neutrophil-to-Albumin Ratio(NPAR, × 10^9^/g), mean	689		0.14 ± 0.07	**<0.001**
Osteoporosis (*N* ≤ -2.5 or *N* ≥ -2.5 ), n (%)	703	Yes	582 (82.79%)	**<0.001**
		No	121 (17.21%)	

Bold fonts indicate P value < 0.05.

**Table 2:** The baseline characteristics of elderly patients with thoracolumbar fractures were included in the final analysis after the missing values were filled in.

Characteristics(N = 703)			Mean or proportion	
	N			p
Age[year], mean	703		75.94 ± 8.71	**0.006**
Gender, n(%)	703	Male	115 (16.36%)	**< 0.001**
		Female	588 (83.64%)	
Height[cm], mean	703		155.18 ± 6.63	**< 0.001**
Weight[kg], mean	703		53.54 ± 10.73	**< 0.001**
BMI[kg/m^2^], mean	703		22.22 ± 4.09	**< 0.001**
Drug allergy, n (%)		Yes	17 (2.42%)	0.252
		No	686 (97.58%)	
Traumatism, n (%)		Yes	293(41.68%)	**< 0.001**
		No	410(58.32%)	
Osteoporosis, n (%)		Yes	527(74.96%)	**0.001**
		No	176(25.04%)	
Hypertension, n (%)		Yes	274(38.98%)	**0.001**
		No	429(61.02%)	
Diabetes, n (%)		YES	83 (11.81%)	**< 0.001**
		No	620 (88.19%)	
Pulmonary disease, n (%)		Yes	55 (7.82%)	0.256
		No	648 (92.18%)	
Kidney disease, n (%)		Yes	10 (1.42%)	**0.002**
		No	693 (98.56%)	
Tumor history, n (%)		Yes	31 (4.41%)	**0.004**
		No	672 (95.59%)	
Erythrocyte sedimentation rate(ESR), mean	703		40.08 ± 24.98	0.418
C-reactive protein(CRP), mean	703		25.85 ± 23.17	0.132
Hemoglobin(HGB)[g/dL], mean	703		120.76 ± 17.88	**< 0.001**
Red blood cell(RBC)[×10^9^/L], mean	703		4.25 ± 0.67	0.487
White blood cell(WBC)[×10^12^/L], mean	703		7.34 ± 2.65	**0.013**
Neutrophil cell(NC)[×10^9^/L], mean	703		5.08 ± 2.47	**0.024**
Lymphocyte cell(LC)[×10^9^/L], mean	703		1.48 ± 0.61	0.543
Monocyte cell(MC)[×109/L], mean	703		10.98 ± 25.66	**0.026**
Platelet(PLT)[×10^9^/L], mean	703		241.06 ± 78.18	**< 0.001**
Fasting blood-glucose(FBG)[mm/h], mean	703		6.2 ± 2.55	**0.001**
Natrium(Na^2+^)[mmol/L], mean	703		140.98 ± 2.79	0.212
Kalium(K+)[mmol/L], mean	703		3.9 ± 0.43	0.296
Chloridion(Cl-)[mmol/L], mean	703		104.94 ± 3.2	0.799
Calcium(Ca^2+^)[mmol/L], mean	703		2.25 ± 0.14	0.056
Urea[mmol/L], mean	703		6.76 ± 3.38	**0.028**
Serum creatinine[umol/L], mean	703		76.6 ± 75.7	**0.001**
Uric acid(Ua)[μmol/L], mean	703		332.12 ± 115.94	**< 0.001**
Total cholesterol(TC)[mmol.L], mean	703		4.73 ± 1.08	0.053
Triglyceride(TG)[mmol.L], mean	703		1.4 ± 0.72	0.118
Albumin(ALB)[g/L], mean	703		37.56 ± 4.6	0.307
Globulin(GLB)[g/L], mean	703		32.93 ± 5.84	0.261
Albumin/Globulin(AG), mean	703		1.18 ± 0.26	133
Total bilirubin(TBil)[μmol/L], mean	703		12.84 ± 8.43	0.171
Direct bilirubin(DBil)[μmol/L], mean	703		4.57 ± 3.07	0.169
L2-BMD, mean	703		-3.71 ± 1.16	**< 0.001**
L3-BMD, mean	703		-3.81 ± 1.49	**< 0.001**
L4-BMD, mean	703		-3.51 ± 1.29	**< 0.001**
TS-BMD, mean	703		-3.68 ± 1.24	**< 0.001**
Platelet-to-Albumin Ratio(PAR)(× 10^9^/g), mean	703		6.52 ± 2.37	**0.009**
Neutrophil-to-Albumin Ratio(NPAR)(×10^9^/g), mean	703		0.14 ± 0.07	**0.005**
Osteoporosis (*N* ≤ -2.5 or *N* ≥ -2.5 ), n (%)	703	Yes	582 (82.79%)	**<0.001**
		No	121 (17.21%)	

Bold fonts indicate P value < 0.05.

### 2.3 Bone mineral density

Given that this study focuses on patients with TLFFs, the BMD measurements included in this research primarily target the spinal region. At our institution, dual-energy X-ray absorptiometry (DXA) serves as the primary modality for BMD assessment. Notably, DXA is globally acknowledged as the “gold standard” for quantitative BMD evaluation and remains the sole reference for the World Health Organization (WHO) in establishing diagnostic criteria for osteoporosis—a status consistently validated in clinical practice and orthopedic research within the osteoporosis field (21). TS-BMD refers to the average bone density of the L2-L4 vertebrae, which is expressed in T-score.

### 2.4 Platelet-to-albumin ratio

The PAR means the ratio between platelets and albumin. We can obtain it by calculating the values included in the study. PAR levels were divided into four groups based on quartile levels: first quartile (Q1): 1.92–4.92 × 10^9^/g, second quartile (Q2): 4.92–6.22 × 10^9^/g, third quartile (Q3): 6.22–7.66 × 10^9^/g, fourth quartile (Q4): 7.66–21.19 × 10^9^/g.

### 2.5 Statistical analysis

Partial covariate missingness was observed in the patient data of the present study. After selecting patients with complete outcome data, missing values for the remaining covariates were imputed. Baseline characteristics of the study participants were described using weighted statistical approaches: categorical variables were presented as weighted proportions, with intergroup comparisons conducted via weighted chi-square tests; continuous variables were expressed as weighted mean ± standard error (SE), and intergroup comparisons were performed using weighted t-tests (22).

Univariate linear regression analysis was performed to initially explore the associations between variables and BMD. Subsequently, after grouping the study variables by quartiles (Q1–Q4), the relationships between baseline characteristics and outcomes were re-evaluated using the same analytical approaches as above; this method facilitated the further identification of which covariates exerted an impact in the present study. For the analysis of associations between study variables and outcomes, a multivariable weighted linear regression model was employed. Similarly, after grouping the study variables by quartiles, the aforementioned analysis was repeated to identify the interval with the most prominent association. The linear regression hypothesis test employed the Shapiro-Wilk test to verify the normality of residuals (P = 0.21), the Levene test to verify the homogeneity of variances (P = 0.35), and the Cook distance test did not identify any abnormal leverage points (all Cook values were less than 1), confirming that all model assumptions were met. The details can be found in **Tables 3** and **4**.

**Table 3:** Comparison of data after 4-category classification using the PAR index.

Characteristics		Data groups (Mean or proportion)					
		Group 1(n=176)	Group 2(n=176)	Group 3(n=176)	Group 4(n=176)	Statistics	P
Age[year], mean		75.48 ± 9.48	75.73 ± 86.68	76.47 ± 8.46	76.09 ± 8.19	0.426	0.734
Gender, n(%)	Male	37 (21.02%)	28 (15.91%)	23 (13.07%)	27 (15.43%)	4.328	0.228
	Female	139 (78.98%)	148 (84.09%)	153 (86.93%)	148 (84.57%)		
Height[cm], mean		156.26 ± 6.36	154.93 ± 6.75	154.82 ± 7.07	154.69 ± 6.24	2.152	0.092
Weight[kg], mean		54.61 ± 11.3	53.94 ± 11.2	54.22 ± 10.4	51.39 ± 9.73	3.266	**0.021**
BMI[kg/m^2^], mean		22.32 ± 4.27	22.4 ± 4	22.63 ± 4.25	21.53 ± 3.75	2.399	0.067
Drug allergy, n (%)	Yes	6 (3.41%)	4 (2.27%)	6 (3.41%)	1 (0.57%)	4.010	0.26
	No	170 (96.59%)	172 (97.73%)	170 (96.59%)	174 (99.43%)		
Traumatism, n (%)	Yes	102 (57.95%)	68 (38.64%)	72 (40.91%)	51 (29.14%)	31.207	**<0.001**
	No	74 (42.05%)	108 (61.36%)	104 (59.09%)	124 (70.86%)		
Osteoporosis, n (%)	Yes	136 (77.27%)	122 (69.32%)	135 (76.7%)	134 (76.57%)	4.014	0.26
	No	40 (22.73%)	54 (30.68%)	41 (23.3%)	41 (23.43%)		
Hypertension, n (%)	Yes	59 (33.52%)	62 (35.23%)	77 (43.75%)	76 (43.43%)	6.386	0.094
	No	117 (66.48%)	114 (64.77%)	99 (56.25%)	99 (56.57%)		
Diabetes, n (%)	YES	21 (11.93%)	14 (7.95%)	21 (11.93%)	27 (15.43%)	4.718	0.194
	No	155 (88.07%)	162 (92.05%)	155 (88.07%)	148 (84.57%)		
Pulmonary disease, n (%)	Yes	11 (6.25%)	10 (5.68%)	14 (7.95%)	20 (11.43%)	4.882	0.181
	No	165 (93.75%)	166 (94.32%)	162 (92.05%)	155 (88.57%)		
Kidney disease, n (%)	Yes	4 (2.27%)	1 (0.57%)	2 (1.14%)	3 (1.70%)	2.127	0.546
	No	172 (97.73%)	175 (99.43%)	173 (98.86%)	173 (98.30%)		
Tumor history, n (%)	Yes	10 (5.68%)	5 (2.86%)	6 (3.41%)	10 (5.68%)	2.356	0.502
	No	166 (94.32%)	170 (97.14%)	170 (96.59%)	166 (94.32%)		
Erythrocyte sedimentation rate(ESR), mean		30.84 ± 20.1	33.96 ± 18.35	43.5 ± 25.88	52.07 ± 28.61	29.229	**<0.001**
C-reactive protein(CRP), mean		24.46 ± 21.8	26.46 ± 23.98	24.46 ± 21.11	28.04 ± 25.56	0.983	0.4
Hemoglobin(HGB)[g/dL], mean		123.33 ± 18.25	122.68 ± 15.56	121.45 ± 16.9	115.57 ± 19.65	7.071	**<0.001**
Red blood cell(RBC)[×10^9^/L], mean		4.3 ± 0.68	4.3 ± 0.63	4.25 ± 0.65	4.14 ± 0.72	2.209	0.086
White blood cell(WBC)[×10^12^/L], mean		6.62 ± 2.36	7.03 ± 2.28	7.52 ± 3.03	8.21 ± 2.63	12.159	**<0.001**
Neutrophil cell(NC)[×10^9^/L], mean		4.63 ± 2.19	4.83 ± 2.15	5.17 ± 2.81	5.69 ± 2.55	6.281	**<0.001**
Lymphocyte cell(LC)[×10^9^/L], mean		1.29 ± 0.61	1.47 ± 0.56	1.52 ± 0.56	1.63 ± 0.66	9.770	**<0.001**
Monocyte cell(MC)[×10^9^/L], mean		8.63 ± 20.35	10.1 ± 21.43	12.86 ± 31.51	12.32 ± 27.73	1.034	0.377
Platelet(PLT)[×10^9^/L], mean		157.84 ± 34.92	216.53 ± 26.03	255.07 ± 30.25	335.32 ± 71.27	493.507	**<0.001**
Fasting blood-glucose(FBG)[mm/h], mean		6.05 ± 2.54	6.13 ± 2.24	6.44 ± 3.01	6.17 ± 2.34	0.775	0.508
Natrium(Na2+)[mmol/L], mean		141.13 ± 2.59	141.25 ± 2.51	140.89 ± 2.64	140.66 ± 3.33	1.563	0.197
Kalium(K^+^)[mmol/L], mean		3.85 ± 0.39	3.89 ± 0.47	3.9 ± 0.41	3.95 ± 0.46	1.645	0.178
Chloridion(Cl-)[mmol/L], mean		105.21 ± 3.14	105.17 ± 2.94	104.85 ± 3.06	104.52 ± 3.59	1.791	0.147
Calcium(Ca^2+^)[mmol/L], mean		2.24 ± 0.14	2.26 ± 0.14	2.25 ± 0.13	2.25 ± 0.16	0.731	0.533
Urea[mmol/L], mean		7.03 ± 4.42	6.67 ± 2.67	6.56 ± 2.45	6.79 ± 3.62	0.628	0.597
Serum creatinine[umol/L], mean		90.66 ± 136.82	72.01 ± 36.83	71.18 ± 32.28	72.54 ± 40.16	2.727	**0.043**
Uric acid(Ua)[μmol/L], mean		332.06 ± 108.06	333 ± 114	334.37 ± 112.4	329.02 ± 129.2	0.067	0.977
Total cholesterol(TC)[mmol.L], mean		4.65 ± 1.18	4.64 ± 0.99	4.86 ± 1.01	4.77 ± 1.13	1.636	0.18
Triglyceride(TG)[mmol.L], mean		1.33 ± 0.69	1.41 ± 0.74	1.45 ± 0.73	1.42 ± 0.72	0.822	0.482
Albumin(ALB)[g/L], mean		39.15 ± 4.46	38.68 ± 4.19	37.03 ± 4.09	35.37 ± 4.69	27.280	**<0.001**
Globulin(GLB)[g/L], mean		31.45 ± 4.96	31.93 ± 4.79	33.59 ± 5.4	34.76 ± 7.31	12.545	**<0.001**
Albumin/Globulin(AG), mean		1.28 ± 0.29	1.24 ± 0.23	1.13 ± 0.22	1.06 ± 0.23	29.682	**<0.001**
Total bilirubin(TBil)[μmol/L], mean		16.27 ± 12.69	12.44 ± 6.9	11.93 ± 5	10.72 ± 5.85	15.069	**<0.001**
Direct bilirubin(DBil)[μmol/L], mean		5.58 ± 3.78	4.34 ± 2.53	4.33 ± 2.77	4.02 ± 2.83	9.186	**<0.001**
L2-BMD, mean		-3.58 ± 1.11	-3.62 ± 1.19	-3.72 ± 1.14	-3.94 ± 1.17	3.465	**0.016**
L3-BMD, mean		-3.69 ± 1.48	-3.68 ± 1.49	-3.78 ± 1.47	-4.08 ± 1.5	2.701	**0.045**
L4-BMD, mean		-3.36 ± 1.29	-3.47 ± 1.31	-3.45 ± 1.27	-3.76 ± 1.27	3.164	**0.024**
TS-BMD, mean		-3.55 ± 1.24	-3.59 ± 1.26	-3.65 ± 1.22	-3.93 ± 1.23	3.341	**0.019**
Osteoporosis (*N* ≤ -2.5 or *N* ≥ -2.5 ), n (%)	Yes	37 (21.02%)	33 (18.75%)	32 (18.18%)	19 (10.86%)	7.162	0.067
	No	139 (78.98%)	143 (81.25%)	144 (81.82%)	156 (89.14%)		

**Table 4:** Association of TS-BMD with PAR, NPAR.

Index	Outcome	Continuous or categories		Model 1^★^				Model 2^※^		
			β	95%CIlow	95%CIupp	P-value	β	95%CIlow	95%CIupp	P-value
PAR	TS-BMD	PAR	- 0.05604	- 0.09472	- 0.01736	**0.005**	- 0.03772	- 0.07228	- 0.00315	**0.032**
		Q1	Reference				Reference			
		Q2	- 0.04456	- 0.30327	0.21416	0.735	0.01887	- 0.21162	0.24937	0.872
		Q3	- 0.10340	- 0.36212	0.15532	0.433	- 0.01166	- 0.24288	0.21955	0.921
		Q4	- 0.37998	- 0.63907	- 0.12089	**0.004**	- 0.25013	- 0.48151	- 0.01874	**0.034**
NPAR	TS-BMD	NPAR	1.75861	- 0.44233	3.07489	**0.009**	0.92366	- 0.26221	2.10953	0.127
		Q1	Reference				Reference			
		Q2	- 0.07057	- 0.32894	0.18781	0.592	- 0.07988	- 0.31263	0.15288	0.501
		Q3	0.25514	- 0.00323	0.51352	0.053	0.14972	- 0.08274	0.38217	0.206
		Q4	0.30509	0.04635	0.56384	**0.021**	0.13582	- 0.09753	0.36918	0.254
Index	Outcome	Continuous or categories	β	Model 3^§^			β	Model 4^#^		
				95%CIlow	95%CIupp	P-value		95%CIlow	95%CIupp	P-value
PAR	TS-BMD	PAR	- 0.04643	- 0.08949	- 0.00338	**0.035**	- 0.05089	- 0.09077	- 0.01099	**0.012**
		Q1	Reference				Reference			
		Q2	0.01321	- 0.21823	0.24465	0.911	0.03061	-0.19784	0.25906	0.793
		Q3	- 0.04314	- 0.28719	0.20090	0.729	- 0.03911	- 0.27537	0.19714	0.745
		Q4	- 0.27867	- 0.54575	- 0.01159	**0.041**	- 0.29503	- 0.54726	- 0.04281	**0.22**
NPAR	TS-BMD	NPAR	2.07705	- 6.52654	10.68064	0.636	6.71879	0.14805	13.2895	**0.045**
		Q1	Reference				Reference			
		Q2	- 0.08320	- 0.32880	0.16241	0.506	- 0.09665	- 0.34281	0.14952	0.441
		Q3	0.16399	- 0.12229	0.45028	0.261	0.12323	- 0.16258	0.40905	0.398
		Q4	0.13290	- 0.28734	0.55315	0.535	0.12891	- 0.28795	0.54577	0.544

PAR: Q1(1.92–4.92 × 10^9^/g), Q2(4.92–6.22 × 10^9^/g), Q3(6.22–7.66 × 10^9^/g), Q4(7.66–21.19 × 10^9^/g);

NPAR: Q1(0.02-0.09 × 10^9^/g), Q2(0.09 - 0.12 × 10^9^/g), Q3(0.12 - 0.16 × 10^9^/g), Q4(0.16 - 0.78 × 10^9^/g);

Bold fonts indicate P value < 0.05.

Model 1^★^: Unadjusted model.

Model 2^※^: Age (≥ 55 years old), Gender (Male or Female), BMI.

Model 3^§^: Age (≥ 55 years old), Gender (Male or Female), BMI, Diabetes (Yes, no), ESR, CRP, HGB, RBC, WBC, NC, LC, MC, PLT, FBG, Na^2+^, K^+^, Cl^-^, Ca^2+^, Urea, Ua, TC, TG, Albumin, Globunin, TBil, DBil

Model 4^#^: Age (≥ 55 years old), Gender (Male or Female), BMI, Diabetes (Yes, no), ESR, CRP, HGB, RBC, WBC, LC, MC, FBG, Na^2+^, K^+^, Cl^-^, Ca^2+^, Urea, Ua, TC, TG, Globunin, TBil, DBil.

TB-BMD, Total spine bone mineral density; PAR, platelet-to-albumin ratio; NPAR, neutrophil-to-albumin ratio.

The missing data were imputed using the multiple imputation method (with 5 imputations). The imputation model included all covariates and the outcome variable. The imputed data were found to have no significant deviations after inspection.Linear regression assumptions were verified: Shapiro-Wilk test confirmed residual normality (p=0.21), Levene test showed homogeneous variances (p=0.35), and Cook distance test identified no influential outliers (all Cook values <1).

All statistical analyses were conducted using IBM SPSS Statistics 17, Stata/SE 18.0, or R 4.0.3 software, with statistical significance defined as a two-tailed P-value < 0.05.

## 3 Results

### 3.1 Baseline characteristics

**Figure 2:**
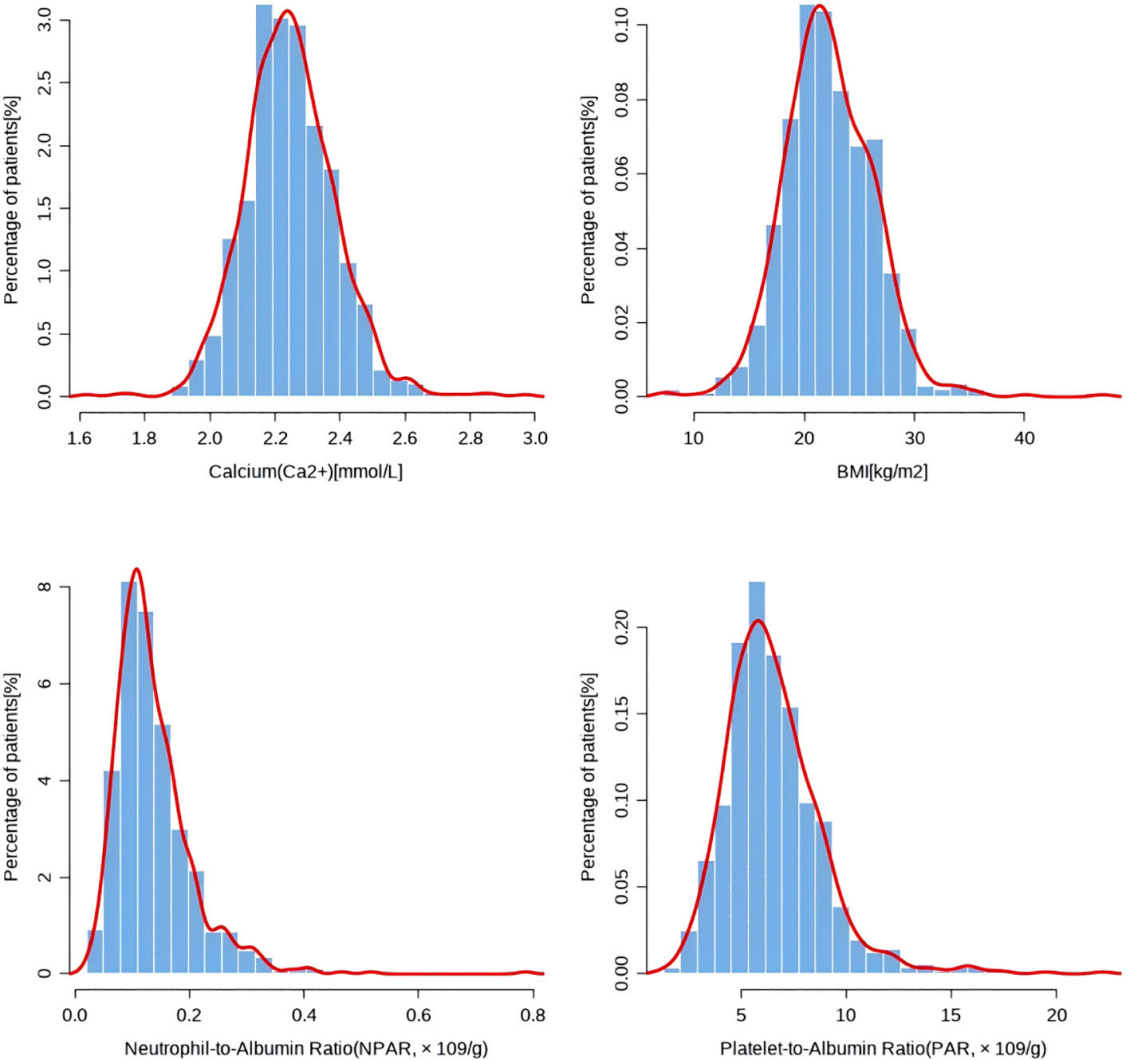
The bar chart of the Calcium, BMD, PAR, NPAR in **Table 1**.

**Table 1** presents the baseline characteristics of elderly patients with thoracolumbar fractures before missing value imputation, while **Table 2** shows the characteristics after mean-based random missing value imputation, with the total sample size remaining 703 patients post-imputation. **Figure 2** presents the distribution of the relevant data through a bar chart.

The mean age of the patients was 75.94 ± 8.71 years(p = 0.006) indicating a potential significant difference in age compared to the reference group. Females accounted for 83.64% (588 cases) of the sample, and males for 16.36% (115 cases) (p < 0.001), suggesting that elderly females are more susceptible to thoracolumbar fractures, which is consistent with the higher incidence of osteoporosis in elderly women. Before imputation, the mean height, weight, and BMI of 445 patients were 155.14 ± 8.03 cm, 53.84 ± 10.41 kg, and 22.3 ± 3.68 kg/m², respectively. After imputation, these values changed to 155.18 ± 6.63 cm, 53.54 ± 10.73 kg, and 22.22 ± 4.09 kg/m² for all 703 patients. All three anthropometric indicators had a p-value < 0.001, indicating clinical relevance.

The prevalence of diabetes was as high as 88.19% (620 cases, p < 0.001), pulmonary disease 92.18% (648 cases), kidney disease 98.72% (693 cases), and tumor history 95.73% (672 cases). Significant p-values were observed for diabetes (p < 0.001), kidney disease (p = 0.002), and tumor history (p = 0.004), implying an association with thoracolumbar fractures, while pulmonary disease showed no significant difference (p = 0.256). Hypertension was present in 61.02% (429 cases, p = 0.001), a history of traumatism in 58.32% (410 cases, p < 0.001), and osteoporosis in 25.04% (176 cases, p = 0.001), all with significant statistical differences. Drug allergy was relatively rare (2.42%, 17 cases, p = 0.252) and showed no significant association with fractures.

After imputation, the mean hemoglobin (HGB) level was 120.76 ± 17.88 g/dL (p < 0.001), and the mean PLT count was 241.06 ± 78.18 × 10^9^/L (p < 0.001). The mean WBC count was 7.34 ± 2.65 × 10¹²/L (p = 0.013), NC count 5.08 ± 2.47 × 10^9^/L (p = 0.024), and the mean MC count 10.98 ± 25.66 × 10^9^/L (p = 0.026), all showing significant differences. In contrast, RBC and LC counts had no significant differences (p = 0.487 and p = 0.543, respectively). The mean FBG level was 6.2 ± 2.55 mm/h (p = 0.001), the mean serum creatinine 76.6 ± 75.7 μmol/L (p = 0.001), the mean Ua 332.12 ± 115.94 μmol/L (p < 0.001) (23), and the mean urea 6.76 ± 3.38 mmol/L (p = 0.028), all with significant differences. Electrolytes such as Na²^+^, K^+^, and Cl^-^ had non-significant p-values (p = 0.212, p = 0.296, and p = 0.799, respectively), and TC and TG also showed no significant differences (p = 0.053 and p = 0.118, respectively).

The mean BMD values at the L2, L3, L4 vertebrae, and TS-BMD were -3.71 ± 1.16, -3.81 ± 1.49, -3.51 ± 1.29, and -3.68 ± 1.24, respectively, with all p-values < 0.001, indicating severe osteoporosis in the patients.

The mean ESR was 40.08 ± 24.98 (p = 0.418), and the mean CRP level was 25.85 ± 23.17 (p = 0.132), showing no significant differences. The mean ALB level was 37.56 ± 4.6 g/L (p = 0.307), the mean GLB 32.93 ± 5.84 g/L (p = 0.261), and the AG ratio 1.18 ± 0.26 (p = 0.133), all with non-significant differences, suggesting no obvious abnormalities in the overall inflammatory and nutritional status of the patients.

### 3.2 Data comparison after 4-category classification using the PAR

No significant differences were observed in age (p = 0.734) or gender distribution (p = 0.228) among the four groups. The mean weight showed a significant difference (p = 0.021), with the lowest value in Group 4 (51.39 ± 9.73 kg). The mean height (p = 0.092) and BMI (p = 0.067) were close to non-significant differences, showing a decreasing trend from Group 1 to Group 4.

There was a significant difference in the proportion of patients with a history of traumatism among the four groups (p < 0.001), increasing from 42.05% in Group 1 to 70.86% in Group 4. No significant differences were found in the prevalence of osteoporosis (p = 0.26), hypertension (p = 0.094), diabetes (p = 0.194), pulmonary disease (p = 0.181), kidney disease (p = 0.546), or tumor history (p = 0.502).

The mean PLT count increased significantly from Group 1 (157.84 ± 34.92 × 10^9^/L) to Group 4 (335.32 ± 71.27 × 10^9^/L) (p < 0.001). The mean HGB level decreased significantly from Group 1 (123.33 ± 18.25 g/dL) to Group 4 (115.57 ± 19.65 g/dL) (p < 0.001). The mean WBC count increased from Group 1 (6.62 ± 2.36 × 10¹²/L) to Group 4 (8.21 ± 2.63 × 10¹²/L) (p < 0.001), the mean NC count from Group 1 (4.63 ± 2.19 × 10^9^/L) to Group 4 (5.69 ± 2.55 × 10^9^/L) (p < 0.001), and the mean LC count from Group 1 (1.29 ± 0.61 × 10^9^/L) to Group 4 (1.63 ± 0.66 × 10^9^/L) (p < 0.001).

The mean ALB level decreased significantly from Group 1 (39.15 ± 4.46 g/L) to Group 4 (35.37 ± 4.69 g/L) (p < 0.001). The mean GLB level increased from Group 1 (31.45 ± 4.96 g/L) to Group 4 (34.76 ± 7.31 g/L) (p < 0.001), while the AG ratio decreased from Group 1 (1.28 ± 0.29) to Group 4 (1.06 ± 0.23) (p < 0.001). The mean TBil level decreased from Group 1 (16.27 ± 12.69 μmol/L) to Group 4 (10.72 ± 5.85 μmol/L) (p < 0.001), and the mean DBil level from Group 1 (5.58 ± 3.78 μmol/L) to Group 4 (4.02 ± 2.83 μmol/L) (p < 0.001). The mean serum creatinine level was highest in Group 1 (90.66 ± 136.82 μmol/L) and lower in the other groups (p = 0.043). No significant differences were observed in FBG, Na²^+^, K^+^, Cl^-^, Ca²^+^, urea, Ua, TC, or TG among the four groups.

**Figure 3:**
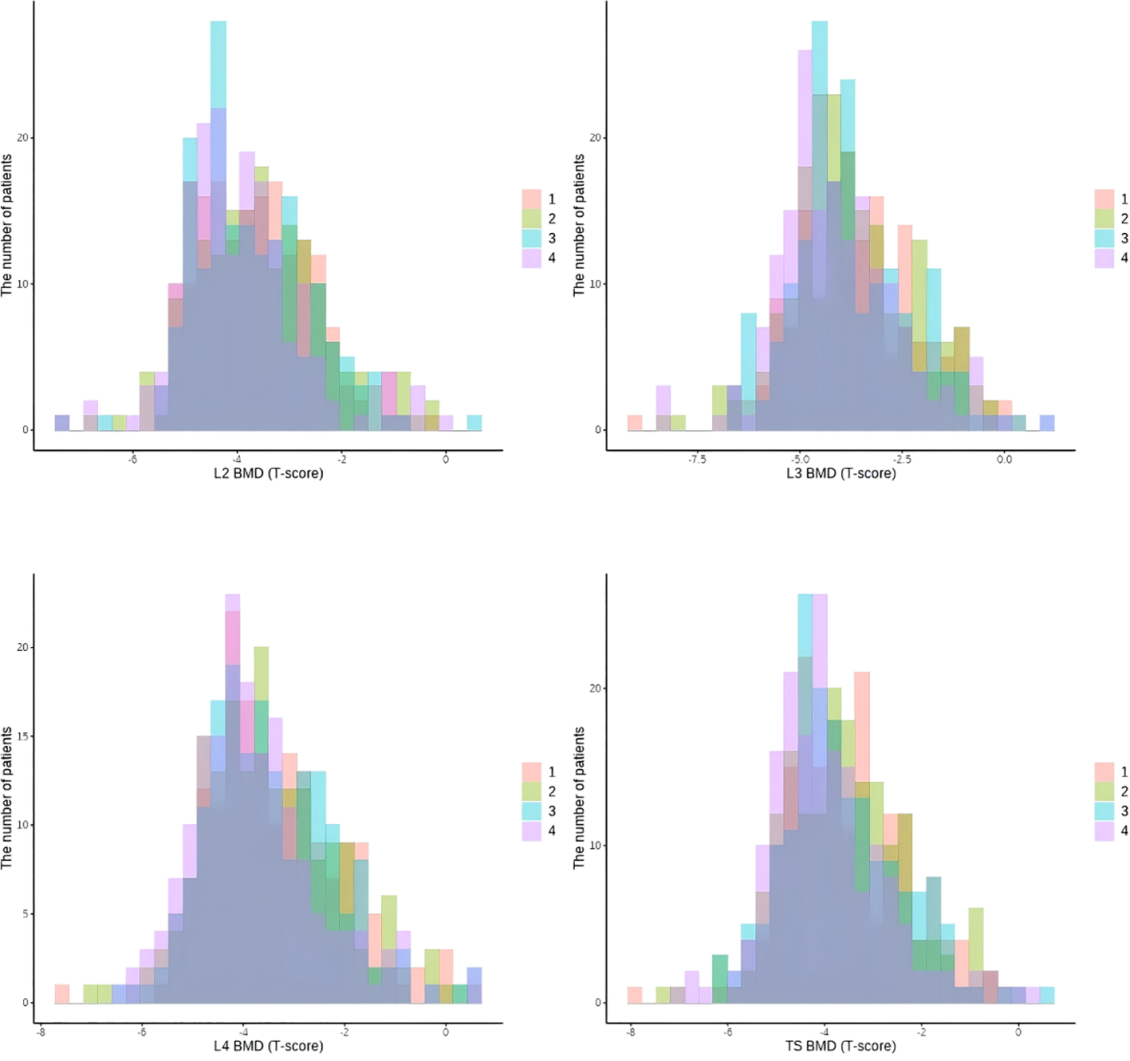
Bar chart of bone density data after 4-category classification using the PAR index.

The mean BMD values at the L2, L3, L4 vertebrae, and TS-BMD showed a decreasing trend from Group 1 to Group 4, with significant differences (p = 0.016, p = 0.045, p = 0.024, and p = 0.019, respectively), and the lowest BMD values were found in Group 4. **Figure 3** shows the distribution of TS-BMD, L2-BMR, L3-BMD, L4-BMD after the PAR grouping.

### 3.3 Association between PAR, NPAR, and total spine BMD

As presented in **Table 3**, per 1-unit increase in PAR correlates with a 0.046 reduction in TS-BMD T-score, based on the World Health Organization (WHO) osteoporosis diagnostic criteria (T-score ≤ -2.5).Tthis equates to an 8% elevated risk of osteoporosis (PMID: 39184075). Furthermore, the TS-BMD T-score in the Q4 group (PAR ≥ 7.66×10^9^/g) was 0.38 lower than that in the Q1 group (PAR ≤ 4.92×10^9^/g), consistent with the clinical consensus that each 0.5 decrease in T-score elevates fracture risk by 15%–20%.

**Table 4** explores the association between the PAR, NPAR, and total spine BMD (TS-BMD) using four different regression models.Continuous PAR Variable: In Model 1 (unadjusted model), the β coefficient for PAR was - 0.05604 (95% CI: - 0.09472 to - 0.01736, p = 0.005), indicating a significant negative association between PAR and TS-BMD. After adjusting for age, gender, and BMI in Model 2, the β coefficient decreased to - 0.03772 (95% CI: - 0.07228 to - 0.00315, p = 0.032), but the significant negative association remained. In Model 3, which further adjusted for a wide range of covariates, including comorbidities, laboratory indicators, and other biochemical markers, the β coefficient was - 0.04643 (95% CI: - 0.08949 to - 0.00338, p = 0.035), still showing a significant negative association. This suggests that an increase in the continuous PAR value is independently associated with a decrease in TS-BMD.

Using Q1 as the reference group, in Model 1, the β coefficient for Q4 was - 0.37998 (95% CI: - 0.63907 to - 0.12089, p = 0.004), indicating a significant negative association between the highest PAR quartile (Q4) and TS-BMD. After adjusting for covariates in Model 2, the β coefficient for Q4 was - 0.25013 (95% CI: - 0.48151 to - 0.01874, p = 0.034), and in Model 3, it was - 0.27867 (95% CI: - 0.54575 to - 0.01159, p = 0.041). The significant negative association persisted in both adjusted models, confirming that patients in the highest PAR quartile have significantly lower TS-BMD compared to those in the lowest quartile (Q1). No significant associations were observed for Q2 or Q3 in any of the models.

In Model 1, the β coefficient for NPAR was 1.75861 (95% CI: - 0.44233 to 3.07489, p = 0.009), indicating a significant positive association between NPAR and TS-BMD. However, after adjusting for age, gender, and BMI in Model 2, the β coefficient decreased to 0.92366 (95% CI: - 0.26221 to 2.10953, p = 0.127), and the association became non-significant. In Model 3, with further adjustment of multiple covariates, the β coefficient was 2.07705 (95% CI: - 6.52654 to 10.68064, p = 0.636), showing no significant association. This suggests that the initial significant positive association between continuous NPAR and TS-BMD in the unadjusted model is not independent and may be confounded by other variables.

Using Q1 as the reference group, in Model 1, the β coefficient for Q4 was 0.30509 (95% CI: 0.04635 to 0.56384, p = 0.021), indicating a significant positive association between the highest NPAR quartile (Q4) and TS-BMD. However, after adjusting for covariates in Model 2, the β coefficient for Q4 was 0.13582 (95% CI: - 0.09753 to 0.36918, p = 0.254), and in Model 3, it was 0.13290 (95% CI: - 0.28734 to 0.55315, p = 0.535). The significant association disappeared in both adjusted models. For Q2 and Q3, no significant associations were observed in any of the models.

To mitigate over-adjustment bias introduced by the components of NPAR and PAR, we constructed regression models (Model 4 in **Table 4**) that either included or excluded PLT, NC, and ALB. Results confirmed a robust, independent negative association between PAR and total spine bone mineral density (TS-BMD): for continuous PAR, the β coefficient was -0.05089 (95% CI: - 0.09077 to - 0.01099, p = 0.012), consistent with the trends observed in Models 1–3 (β: - 0.03772 to - 0.05604, all p = 0.032). When PAR was stratified into quartiles (Q1 as the reference), only the highest quartile (Q4) exhibited a negative association with TS-BMD (β = - 0.2903, 95% CI: - 0.54726 to - 0.04281), aligning with the significant negative associations of Q4 in Models 1–3 (β: - 0.25013 to - 0.37998, all p < 0.034); Q2 and Q3 showed no meaningful associations. These findings confirm that the negative link between PAR and TS-BMD is primarily driven by continuous PAR and the highest PAR quartile, and this relationship remains stable even after addressing over-adjustment bias related to PLT, NC, and ALB.

In contrast, the association between NPAR and TS-BMD lacked consistency: while Model 4 yielded a significant positive β coefficient for continuous NPAR (6.71879, 95% CI: 0.14805 to 13.2895, p = 0.045), this contradicted the non-significant results in Models 2–3 (β: 0.92366 and 2.07705, both p > 0.12). None of the NPAR quartile groups (Q2, Q3, Q4) showed a statistically significant association with TS-BMD (p > 0.39 for all), consistent with adjusted Models 2–3. These results indicate that the initial association between NPAR and TS-BMD is confounded by covariates, and its stability is substantially weaker than that of the PAR-TS-BMD association.

### 3.4 Association between PAR, NPAR, and osteoporosis

We further classified total spine BMD (TS-BMD) into a binary outcome (osteoporosis vs. non-osteoporosis) per WHO diagnostic criteria (TS-BMD ≤ −2.5), and used logistic regression to explore associations between PAR, NPAR, and osteoporosis (**Table 5**). For PAR, consistent significant associations with osteoporosis were observed: in the unadjusted Model 1, the β coefficient for PAR was 0.13608 (p = 0.006), indicating that higher PAR correlated with increased osteoporosis risk; this association remained significant after adjusting for age, sex, and BMI (Model 2: β = 0.12139, p = 0.021). While adjustment for a broader set of covariates in Model 3 attenuated this link to non-significance (β = −0.10985, p = 0.762), Model 4—designed to mitigate over-adjustment bias from PAR/NPAR components—restored a significant positive association (β = 0.16587, p = 0.013), confirming the stable predictive value of PAR for osteoporosis.

**Table 5:** Association of osteoporosis (*N* ≤ -2.5 or *N* ≥ -2.5) with PAR, NPAR.

Index	Outcome	Continuous or categories					
			β	z	95%CIlow	95%CIupp	P-value
PAR	Osteoporosis	**Model 1^★^**	0.13608	2.74	0.03883	0.23333	**0.006**
		**Model 2^※^**	0.12139	2.31	0.80632	0.61927	**0.021**
		**Model 3^§^**	- 0.10985	- 0.30	- 0.81943	0.59974	0.762
		**Model 4^#^**	0.16587	2.49	0.03551	0.29622	**0.013**
Index	Outcome	Continuous or categories	β	z	95%CIlow	95%CIupp	P-value
								NPAR	Osteoporosis	**Model 1^★^**	-1.94747	- 1.48	- 0.52716	0.63221	0.139
	**Model 2^※^**	- 0.45096	- 0.28	- 3.55908	2.65716	0.776		
	**Model 3^§^**	9.67492	0.76	- 15.30728	34.65712	0.448		
	**Model 4^#^**	- 0.51776	- 0.09	- 11.89015	10.85463	0.929		

PAR: Q1(1.92–4.92 × 10^9^/g), Q2(4.92–6.22 × 10^9^/g), Q3(6.22–7.66 × 10^9^/g), Q4(7.66–21.19 × 10^9^/g);

NPAR: Q1(0.02-0.09 × 10^9^/g), Q2(0.09 - 0.12 × 10^9^/g), Q3(0.12 - 0.16 × 10^9^/g), Q4(0.16 - 0.78 × 10^9^/g);

Bold fonts indicate P value < 0.05.

Model 1^★^: Unadjusted model.

Model 2^※^: Age (≥ 55 years old), Gender (Male or Female), BMI.

Model 3^§^: Age (≥ 55 years old), Gender (Male or Female), BMI, Diabetes (Yes, no), ESR, CRP, HGB, RBC, WBC, NC, LC, MC, PLT, FBG, Na^2+^, K^+^, Cl^-^, Ca^2+^, Urea, Ua, TC, TG, Albumin, Globunin, TBil, DBil

Model 4^#^: Age (≥ 55 years old), Gender (Male or Female), BMI, Diabetes (Yes, no), ESR, CRP, HGB, RBC, WBC, LC, MC, FBG, Na^2+^, K^+^, Cl^-^, Ca^2+^, Urea, Ua, TC, TG, Globunin, TBil, DBil.

TB-BMD, Total spine bone mineral density; PAR, platelet-to-albumin ratio; NPAR, neutrophil-to-albumin ratio.

In contrast, NPAR showed no meaningful association with osteoporosis across all models: β coefficients for NPAR in Models 1–4 were non-significant (all p > 0.139), indicating that NPAR is not an independent predictor of osteoporosis.

Together, these findings—from both continuous TS-BMD and binary osteoporosis analyses—demonstrate that PAR exhibits a robust negative correlation with TS-BMD and a significant positive association with osteoporosis risk, positioning it as a potential key marker of bone health outcomes. In contrast, NPAR shows weak, inconsistent links to bone health endpoints, limiting its clinical utility in this context.

## 4 Discussion

### 4.1 Baseline characteristics: clinical & research implications

The baseline features of 703 elderly patients with thoracolumbar fractures offer critical insights into the clinical profile of this population. The high mean age (75.94 ± 8.71 years) aligns with the established epidemiology of thoracolumbar fractures, which are more prevalent in the elderly due to age-related declines in bone density and muscle mass (osteosarcopenia). The notable gender imbalance—with females representing 83.64% of the sample—corresponds to the higher incidence of osteoporosis in postmenopausal women, a key risk factor for fragility fractures (24, 25). This underscores the necessity of gender-specific prevention and treatment strategies for elderly individuals at risk of thoracolumbar fractures.

The high prevalence of comorbidities, including diabetes, kidney disease, and a history of tumors, is striking. Diabetes is known to disrupt bone metabolism, reducing bone strength and elevating fracture risk (13, 26). Chronic kidney disease (CKD) is linked to renal osteodystrophy, a condition characterized by abnormal bone mineralization that further exacerbates bone loss in the elderly (27–29). A tumor history—particularly for tumors involving bone or treated with chemotherapy/radiotherapy—can also exert adverse effects on bone health. These comorbidities complicate the management of thoracolumbar fractures, as they may increase the risk of perioperative complications and impair fracture healing (30). In these cases of the current study, we did not find the aforementioned association with the outcome. Further research on specific populations may be necessary. Clinicians should therefore conduct a comprehensive assessment of comorbidities to develop individualized treatment plans for elderly patients with thoracolumbar fractures.

Laboratory indicators reveal several notable findings. Significant reductions in HGB and ALB levels, coupled with increases in WBC, NC, and MC counts, suggest the presence of chronic inflammation and malnutrition in a subset of patients. Chronic inflammation promotes bone resorption by activating osteoclasts, thereby contributing to osteoporosis and elevated fracture risk (22). Malnutrition—especially protein deficiency, reflected by low ALB levels—can hinder bone formation and muscle function, further increasing fracture risk and delaying recovery. These observations emphasize the importance of evaluating and addressing inflammatory and nutritional status in the management of elderly patients with thoracolumbar fractures (14).

The severely reduced BMD values at the lumbar spine (L2-L4) and TS-BMD confirm the high prevalence of osteoporosis in this cohort. The mean BMD values (ranging from -3.51 to 3.81) are well below the WHO threshold for osteoporosis (T-score ≤ -2.5), indicating a high risk of future fractures (21). This highlights the need for early diagnosis and treatment of osteoporosis in elderly patients with thoracolumbar fractures to prevent subsequent fractures (24, 31).

### 4.2 PAR: insights into disease pathophysiology

The 4-category classification based on the PAR provides valuable insights into the association between PAR and various clinical and laboratory parameters. The significant increase in PLT count and decrease in ALB level from Group 1 to Group 4 validate the PAR index as a composite marker of platelet activation and nutritional status. Platelets play a crucial role in the inflammatory response and bone metabolism. Activated platelets release a range of cytokines and growth factors, such as transforming growth factor-β (TGF-β) and platelet-derived growth factor (PDGF), which can regulate osteoclast and osteoblast activity (32, 33).TGF-β exerts a dual detrimental effect on bone homeostasis by promoting osteoclast differentiation while suppressing osteoblast activity, as evidenced in relevant studies. This dual action disrupts the bone formation-resorption balance, driving pathological bone loss and osteoporosis progression (34, 35). Elevated PLT counts may thus contribute to increased bone resorption and reduced bone density, as observed in the higher PAR groups.

The progressive significant decrease in HGB levels from Group 1 to Group 4 suggests a potential association between PAR and anemia. As a key nutritional biomarker, albumin correlates closely with protein intake; reduced albumin levels indicate insufficient protein supply, which directly impairs osteoblast activity and hinders bone matrix synthesis, thereby compromising bone formation and exacerbating bone metabolism imbalance (36). Notably, anemia is highly prevalent in the elderly population and has been firmly linked to decreased bone mineral density and elevated fracture risk. Underlying mechanisms may involve chronic inflammation, which not only suppresses erythropoiesis but also promotes excessive bone resorption, as well as shared risk factors including malnutrition and chronic diseases (37, 38). Further investigations are warranted to elucidate the causal relationships between PAR, anemia, hypoproteinemia, and bone health.

The significant increase in WBC and NC counts from Group 1 to Group 4 indicates a potential association between PAR and chronic inflammation. Chronic inflammation is a key driver of age-related bone loss and osteoporosis (22). Another study has shown that chronic inflammation leads to excessive bone resorption by activating the NF-κB pathway (39): continuous pro-inflammatory stimulation upregulates NF-κB signaling, promoting the differentiation, maturation and activation of osteoclasts, enhancing the bone resorption capacity of osteoclasts, while inhibiting bone formation mediated by osteoblasts, thereby disrupting the balance of bone and promoting the progression of osteoporosis.

### 4.3 Association between PAR, NPAR, and TS-BMD

The significant negative association between PAR and TS-BMD—which persisted after adjusting for multiple covariates—highlights the potential clinical value of PAR as a predictor of bone density in elderly patients with thoracolumbar fractures. This independent association suggests that PAR can provide additional information beyond traditional risk factors such as age, gender, BMI, and comorbidities. This is particularly relevant in clinical practice, where simple, cost-effective markers are needed to identify patients at high risk of osteoporosis and subsequent fractures.

The finding that patients in the highest PAR quartile (Q4) had significantly lower TS-BMD than those in the lowest quartile (Q1) suggests that PAR can be used to stratify patients based on bone health status. Patients with high PAR values may require more aggressive osteoporosis treatment—such as bisphosphonates or denosumab—to reduce the risk of future fractures (40). Additionally, monitoring PAR levels during follow-up may help assess treatment response and identify patients at risk of treatment failure.

In contrast, the initial significant positive association between the NPAR and TS-BMD in the unadjusted model was not sustained after covariate adjustment, indicating that NPAR is not an independent predictor of bone density. This suggests that the association between NPAR and TS-BMD may be confounded by variables such as age, gender, BMI, or comorbidities. The NPAR index may therefore be less useful than PAR for predicting bone health in elderly patients with thoracolumbar fractures.

The mechanisms underlying the association between PAR and bone density are likely multifactorial. Platelet activation can release pro-inflammatory cytokines and growth factors that promote bone resorption (41, 42). Low ALB levels— a component of the PAR index—indicate malnutrition, which can impair bone formation and muscle function. Chronic inflammation—often associated with elevated platelet counts and low ALB levels—can also contribute to bone loss by activating osteoclasts (11, 13, 14, 32). The combination of these factors may explain the negative association between PAR and bone density observed in this study.

Emerging evidence from studies (39, 43, 44) highlights the superior value of the inflammation-nutrition composite indicator PAR in osteoporosis assessment. Unlike single biomarkers that merely reflect isolated inflammatory or nutritional status, PAR integrates both dimensions, which are closely intertwined with the pathological processes of osteoporosis—chronic inflammation impairs osteoblastic activity and promotes osteoclastic resorption, while malnutrition undermines bone matrix synthesis and mineralization. This composite nature endows PAR with higher sensitivity and specificity in identifying individuals at high risk of osteoporosis, enabling earlier detection of subclinical bone loss compared to conventional single indices. Additionally, PAR exhibits good accessibility as it can be derived from readily available clinical parameters, facilitating its application in routine clinical settings. To further enhance clinical utility, future research should focus on integrating PAR with traditional risk factors such as age, gender, and BMI to construct a comprehensive predictive model. Such a model is expected to improve the accuracy of osteoporosis risk stratification and personalized intervention planning, addressing the limitations of current assessment tools that often rely solely on either composite indicators or traditional factors.

### 4.4 Limitations and future directions

As a retrospective cross-sectional study, this research only explores the association between PAR and TS-BMD and cannot infer causal relationships. PAR may serve as a biological marker reflecting osteoporosis severity, rather than a direct mechanistic factor driving bone loss—prospective studies are needed to verify the temporal sequence and causal links. This study has several limitations that should be considered when interpreting the results. First, it is a cross-sectional study, which limits the ability to establish causal relationships between PAR, NPAR, and bone density. Prospective studies are needed to determine whether high PAR values predict future declines in bone density and increased fracture risk. Second, the PAR and NPAR indices were calculated based on a single measurement, and the variability of these indices over time was not assessed. Future studies should include repeated measurements to evaluate the stability of PAR and NPAR and their association with changes in bone density over time.

Although this study adjusted for multiple covariates, there may still be residual confounding (such as unmeasured nutritional factors, exercise habits, drug use history, etc.) that could affect the strength of the association between PAR and TS-BMD.

Despite these limitations, the findings have important implications for clinical practice and future research. The PAR index may serve as a simple, cost-effective marker for identifying elderly patients with thoracolumbar fractures who are at high risk of severe osteoporosis and subsequent fractures. Further studies are needed to validate the utility of PAR in larger, more diverse populations and to explore the underlying mechanisms of the association between PAR and bone health. Additionally, interventional studies are required to determine whether targeting PAR-related pathways—such as reducing chronic inflammation or improving nutritional status—can enhance bone density and reduce fracture risk in elderly patients with thoracolumbar fractures.

Future research can focus on the following aspects: Firstly, prospective cohort studies to track the long-term association between PAR changes and bone density decline as well as fracture occurrence. Secondly, interventional studies to explore the effects of improving nutritional status (increasing albumin levels) or regulating platelet activity on bone density in elderly patients with osteoporosis. Thirdly, multicenter validation studies to expand the sample size and include populations from different regions and ethnic groups to verify the universality of PAR. Fourthly, construction of a bone loss risk prediction model including PAR by combining machine learning to enhance its clinical application value.

## 5 Conclusion

In the present study, we identified a robust association between the PAR and TS-BMD, with a significant negative correlation observed (**Table 3**). Clinically, platelets and albumin are readily measurable blood-based biomarkers—routinely assessed in standard laboratory panels—rendering the PAR index highly accessible and straightforward to compute. This simplicity underscores its potential utility as a pragmatic tool in clinical practice. Importantly, our findings raise the intriguing possibility that PAR could serve as a reference for assessing the degree of osteoporosis in patients with TLFF.

## Data Availability

The raw data supporting the conclusions of this article will be made available by the authors, without undue reservation.
